# A quadriphasic mechanical model of the human dermis

**DOI:** 10.1007/s10237-024-01827-5

**Published:** 2024-03-15

**Authors:** David Sachs, Raphael Jakob, Gaetana Restivo, Jürg Hafner, Nicole Lindenblatt, Alexander E. Ehret, Edoardo Mazza

**Affiliations:** 1 Institute for Mechanical Systems, ETH Zürich, Zurich, Switzerland; 2 Department of Dermatology, University Hospital Zürich, Zurich, Switzerland; 3 Department of Plastic Surgery and Hand Surgery, University Hospital Zürich, Zurich, Switzerland; 4 Swiss Federal Laboratories for Materials Science and Technology, Experimental Continuum Mechanics, Dübendorf, Switzerland

**Keywords:** Dermis, Mechanobiology, Electrical potential, Osmotic pressure, Quadriphasic model

## Abstract

The present study investigates the multiphasic nature of the mechanical behavior of human dermis. Motivated by experimental observations and by consideration of its composition, a quadriphasic model of the dermis is proposed, distinguishing solid matrix components, interstitial fluid and charged constituents moving within the fluid, i.e., anions and cations. Compression and tensile experiments with and without change of osmolarity of the bath are performed to characterize the chemo-mechanical coupling in the dermis. Model parameters are determined through inverse analysis. The computations predict a dominant role of the permeability in the determination of the temporal evolution of the mechanical response of the tissue. In line with the previous studies on other tissues, the analysis shows that an ideal model based on Donnan’s equilibrium overestimates the osmotic pressure in skin for the case of very dilute solutions. The quadriphasic model is applied to predict changes in dermal cell environment and therefore alterations in what is called the “mechanome,” associated with skin stretch. The simulations indicate that skin deformation causes a variation in several local variables, including in particular the electric field associated with a deformation-induced non-homogeneous distribution of fixed charges.

**Supplementary Information:**

The online version contains supplementary material available at 10.1007/s10237-024-01827-5.

## Introduction

**Fig. 1:**
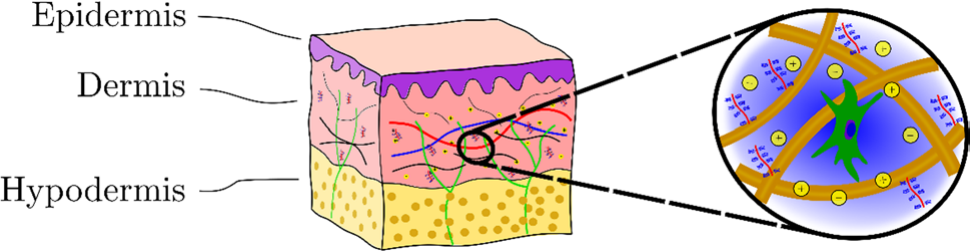
Human skin is composed of three layers. The dermal layer is a dense mesh of collagen and elastin fibers with fixed charges attached to it and interstitial fluid in-between

The three main layers of the human skin are epidermis, dermis and hypodermis. The epidermis is the outermost layer. It is composed of several sheets of keratinocytes and forms a firm barrier to protect the human body against chemical and physical hazard from the environment (Ruth and Freinkel 2001). The hypodermis, the innermost layer, consists of adipose cells connected by a fibrillar network and provides a cushion against pressure and environmental temperature changes (Comley and Fleck 2010). The dermis is situated between these two layers. It is about 1–3 mm thick, and its extracellular matrix is composed of collagen and elastin fibers, which provide skin with its tensile strength and elasticity (Uitto et al. 1989). Proteoglycans and glycosaminoglycans, long polysaccharide chains, are crosslinked to the matrix and provide it with an overall negative charge which causes tissue hydration (Lee et al. 2016). The remaining extracellular space is filled by interstitial fluid, which provides resident cells with nutrients. There is a continuous flow of interstitial fluid as it leaks from the blood capillaries into the interstitial space. It thereby cleans the interstitial space by dragging metabolic waste, which is then absorbed together with the interstitial fluid by the lymphatic network (Swartz and Fleury 2007). Negatively and positively charged ions are present in the interstitial fluid and their distribution, together with the fixed charges of the solid matrix, satisfy the electroneutrality of the extracellular space (Wiig et al. 2000) (Fig. 1).

Cells residing in the dermis feel and react to mechanically induced changes of their surrounding matrix and the interstitial fluid. Skin tension generates stretch of the tissue, which induces changes in various chemo-physical quantities at the microscale, that potentially influences cell behavior. Among the components of what is called the “mechanome” of soft collagenous tissues (Wang et al. 2014), tissue deformation affects the fiber network and thus the cell perceived stiffness. We have recently shown that stretch of soft collagenous tissue can also induce significant displacement of the interstitial fluid associated with variation of its velocity, as well as osmotic and hydrostatic pressures (Ehret et al. 2017; Stracuzzi et al. 2022). As reported for cartilage (Gu et al. 1999; Mow et al. 1999), matrix deformation and interstitial fluid flow can influence ion concentration, leading to alterations of the electric field (Bassett and Pawluk 1972; Grodzinsky et al. 1978; Lai et al. 2000). Deviations from equilibrium of each of these quantities is expected to affect the biological response of cells. Increased stiffness in a stretched collagen network was reported to increase cell proliferation (Discher et al. 2005; Hadjipanayi et al. 2009). Increases in the velocity of fluid shear flow by few $\frac{\mathrm{\mu m}}{{\text{s}}}$ were found to cause in cell alignment and enhanced proliferation (Ng et al. 2005; Ng and Swartz 2003). Electrical and chemical signals can guide cell migration and division, e.g., in wound healing and scars (Thrivikraman et al. 2018; Wolf et al. 2022; Zajdel et al. 2021). In fact, electrical field of gradients of only 0.5 mV across a cell have been shown to induce cell migration (Simpson et al. 2017; Song et al. 2002; Tai et al. 2018; Zhao 2009).

While the experimental quantification of these cues is highly challenging, computational models can provide insights on magnitude and time scales of these biophysical processes. In the past two decades, computational analysis of soft biological tissues advanced from single-phase to multi-phase models, which incorporate osmotic and ionic effects into nonlinear dissipative mechanical representation of tissues (Ateshian and Weiss 2013; J. M. Huyghe and Janssen 1997; Lai et al. 1991, 2000; Sun et al. 1999). These so-called quadriphasic models include the solid displacement, fluid flow and the flow of positively and negatively charged ions in their governing equations. They have been applied to study chemo-mechanical processes in tissues physiologically exposed to compressive forces, such as articular cartilage (Mow and Guo 2003; Wilson, Van Donkelaar, Van Rietbergen, et al., 2005) and the intervertebral disk (Frijns et al. 1997; Schroeder et al. 2006).

No quadriphasic model has been developed for the dermis so far. The relevance of such model representations is provided not only by the fact that skin is physiologically exposed to compression but also by the mechanism of interstitial fluid displacement and tissue dehydration associated with in-plane stretch as previously described for soft collagenous tissues (Ehret et al. 2017). Early experimental contributions investigated the biphasic nature of skin (Oomens et al. 1987), and the relevance of interstitial fluid displacement in skin was confirmed in recent experimental work (Oftadeh et al. 2018; Wahlsten et al. 2019; Woessner et al. 2021). However, only few biphasic model formulations exist for skin. Examples are the model by Zhang et al. which was developed to study drug delivery (Zhang et al. 2009) and latest work to simulate subcutaneous injections (de Lucio et al. 2023; Leng et al. 2021). In our own contributions, we recently investigated experimentally and numerically the interplay of mechanical stretch and chemical signals in human skin (Wahlsten et al. 2019), proposing also a multilayer biphasic model (Sachs et al. 2021) which incorporates osmotic effects based on the assumption of Donnan’s equilibrium (Donnan 1911).

This previously introduced biphasic model (Sachs et al. 2021) successfully captured the inverse poroelastic behavior of skin in ex vivo monotonic uniaxial tension and the time-dependent response observed in vivo through suction experiments. The model, however, assumes an instantaneous equilibrium of ion concentrations, which represents a well-justified simplification when focusing on the mechanical behavior of the tissue (Lanir 1987; Wilson et al. 2005a, b). Accounting for ion flow is required in order to describe electrochemical cues associated with skin stretch and thus for a more complete representation of the mechanome of the human dermis. This is the objective of the present work. To this end, we introduce a quadriphasic model and ensure its consistency with our previously performed measurements (Wahlsten et al. 2019). Additionally, we conducted new experiments to enrich the set of data available to calibrate the model. Specifically, we conducted displacement-controlled and force-controlled compression experiments during which we change the ionic concentration of the external bath. With the new model formulation, we also addressed limitations of our previous one related to the dissipation behavior of the solid component, which was overestimated when compared to experimental data for short observation times and underestimated for longer time scales. To this end, an additional dissipative term is included in the new formulation, which allows to distinguish between fast and slow dissipation of the fibers.

This paper is structured as follows. We first introduce the quadriphasic governing equation for human dermis as a charged hydrated nonlinear viscoelastic tissue. The methods for the new compression experiments are described next, and all experimental results are presented, which were used for model calibration. Corresponding results are reported showing a good agreement between experiments and model predictions. The results of the inverse analysis further point at the non-ideal behavior of the osmotic pressure for very dilute external bath solutions and at the key influence of tissue permeability on the transient mechanical response of skin. Finally, based on the new model, we assess magnitude and distribution of the electrical potential associated with deformations of human skin.

## Material and methods

The dermis is the mechanically dominant layer of human skin (Guimberteau et al. 2017; Meigel et al. 1977). Hence, the present model focuses on this region of the skin, thus combining papillary and reticular dermis into one single layer. The epidermis was shown to contribute to the mechanical response of human skin only marginally in tensile experiments (Wahlsten 2023) and is thus not considered explicitly as a distinct layer but incorporated to reflect its influence on the boundary conditions. In fact, since the epidermis forms a nearly impermeable layer, a no-flow boundary condition is applied to the epidermal surface. The hypodermis was carefully removed in all experiments considered in this work and is thus not represented in the present model.

### Quadriphasic model of the dermis

#### Governing variables and equations

The quadriphasic model is based on the previous multiphase formulations for biological tissues (Ateshian et al. 2013; Ehlers et al. 2009; J. M. Huyghe and Janssen 1997; Lai et al. 1991; Mauck et al. 2003; Stracuzzi et al. 2018; Sun et al. 1999). The system of nonlinear equations is solved for the solid displacements ${\varvec{u}}$, the chemical potential of the fluid ${\mu }_{{\text{f}}}$ as well as the electrochemical potentials of cations ${\mu }_{+}$ and anions ${\mu }_{-}$. The solid displacement ${\varvec{u}}$ is defined through the deformation gradient ${\varvec{F}}={{\varvec{F}}}_{{\text{s}}}=\frac{\partial {{\varvec{\upchi}}}_{{\text{s}}}\left({\mathbf{X}}_{{\text{s}}},t\right)}{\partial {\mathbf{X}}_{{\text{s}}}}$ with determinant $J={\text{det}}({\varvec{F}})$>0, where the function ${{\varvec{\upchi}}}_{s}\left({{\varvec{X}}}_{s},t\right)$ maps the material position ${{\varvec{X}}}_{{\text{s}}}$ to the spatial position ${{\varvec{x}}}_{{\text{s}}}$ of the solid. The chemical potential of the fluid (per unit of mass) is the variable governing fluid motion and is given by Richards and Dover, 1980

1 $${\mu }_{\text{f}}=\frac{p}{{\rho }_{\text{f}}}-\frac{RT}{{\rho }_{\text{f}}}\left({c}_{+}+{c}_{-}\right)+{\mu }_{\text{f,0}},$$

wherein $p$ is the hydrostatic pressure, $R$ is the universal gas constant, $T$ describes the absolute temperature, ${c}_{+}$ and ${c}_{-}$ are the cation and anion concentration in the fluid, ${\rho }_{f}$ is the density of the fluid and ${\mu }_{f,0}$ is the reference chemical potential. The second term in equation (1) describes the osmotic pressure.

The flow of ions depends on the electrochemical potentials of the anions and the cations which read as follows (J. M. Huyghe and Janssen 1997; Richards and Dover, 1980):

2 $${\upmu }_{+}=\frac{{\text{RT}}}{{M}_{+}}{\text{ln}}\left({c}_{+}\right)+\frac{F\Psi }{{M}_{+}}+{\mu }_{+,0},$$

3 $${\mu }_{-}=\frac{{\text{RT}}}{{M}_{-}}{\text{ln}}\left({c}_{-}\right)-\frac{F\Psi }{{M}_{-}}+{\mu }_{-,0}.$$

Therein ${\mu }_{+,0}$ and ${\mu }_{-,0}$ are their reference chemical potentials, $F$ is the Faraday constant, ${M}_{+}$ and ${M}_{-}$ are the molar mass of cations and anions and $\Psi$ is the electrical potential (Guggenheim 1930). The electrical potential describes the work required to move an electrically charged particle, such as an ion, within an electric field (Richards and Dover, 1980). When no external electrical field is applied, the electrical potential solely stems from electrical currents inside of the tissue leading to differences in the ions’ density. These currents can be induced by fluid dragging ions along with it and by diffusion of ions (Lai et al. 2000). From Eqs. (2) and (3), the electrical potential can be related to the internal ion concentrations and their chemical potentials (Eqs. (2 and 3)) under the assumption of ideal behavior by Richards and Dover, 1980.

4 $$\Psi =\frac{{\text{RT}}}{2F}\left[{\text{ln}}\left(\frac{{c}_{-}}{{c}_{+}}\right)+\frac{{M}_{+}}{{\text{RT}}}\left({\mu }_{+}-{\mu }_{+,0}\right)-\frac{{M}_{-}}{{\text{RT}}}\left({\mu }_{-}-{\mu }_{-,0}\right)\right]$$

As explained in Supplementary Sect. 1.1, the concentrations of cations and anions (${c}_{+}\; {\text{and}}\; {c}_{-}$) can be expressed as a function of the corresponding chemical potentials (equations A 10 and A 11). Thus, the electrical potential (Eq. (4)) can be calculated based on the values of the ions’ chemical potential. The same applies to the following equations in which ${c}_{+}$ and ${c}_{-}$ are used for brevity, instead of explicitly providing the corresponding expressions as function of ${\mu }_{+}$ and ${\mu }_{-}$.

The flux of the fluid relative to the solid is defined by ${{\varvec{j}}}_{\text{f}}={\varphi }_{\text{f}}({{\varvec{v}}}_{\text{f}}-{{\varvec{v}}}_{{\text{s}}})$. Therein ${\varphi }_{{\text{f}}}$ is the fluid volume fraction in the current configuration, and ${{\varvec{v}}}_{{\text{f}}}$ and ${{\varvec{v}}}_{{\text{s}}}$ are the spatial velocities of the fluid and the solid phase. The flux is modeled by the extended Darcy’s law (J. Huyghe and Janssen 1999; Sun et al. 1999).

5 $${{\varvec{j}}}_{f}=-{\varvec{k}}\left[{\rho }_{f }\mathrm{ grad }\left({\mu }_{f}\right)+{M}_{+}{c}_{+} {\text{grad}} \left({\mu }_{+}\right)+{{M}_{-}c}_{-}{\text{grad}}\left({\mu }_{-}\right)\right],$$

with ${\varvec{k}}$ being the permeability tensor (Frijns et al. 1998), also referred to as hydraulic conductivity. The second and third terms of Eq. (5) result from the exchange of momentum due to the relative velocity of the corresponding ionic species and the fluid phase under the assumption of Onsager’s reciprocity relationship (Onsager 1931a, 1931b). The permeability tensor was chosen spatially isotropic with a simple deformation dependency (Ateshian and Weiss 2010; Markert 2007)

6 $${\varvec{k}}={k}_{0}{\left(\frac{J-{\varphi }_{s}^{{\text{ref}}}}{1-{\varphi }_{s}^{{\text{ref}}}}\right)}^{\kappa }{\varvec{I}},$$

with $\kappa >0$ being a material parameter and ${k}_{0}$ and ${\varphi }_{\text{s}}^{\text{ref}}$ denoting the permeability and the solid volume fraction in the reference configuration, respectively. The molar fluxes of the cations ${{\varvec{n}}}_{+}={\varphi }_{\text{f}}{c}_{+}\left({{\varvec{v}}}_{+}-{{\varvec{v}}}_{\text{s}}\right)$ and anions ${{\varvec{n}}}_{-}={\varphi }_{\text{f}}{c}_{-}({{\varvec{v}}}_{-}-{{\varvec{v}}}_{\text{s}})$ are prescribed by the diffusion convection equations (J. Huyghe and Janssen 1999; Sun et al. 1999)

7 $${{{\varvec{n}}}_{+}=c}_{+}{{\varvec{j}}}_{\text{f}}-\frac{{M}_{+}{\varphi }_{\text{f}}{c}_{+}{D}_{+}}{{\text{RT}}}{\text{grad}}\left({\mu }_{+}\right),$$

8 $${{{\varvec{n}}}_{-}=c}_{-}{{\varvec{j}}}_{f}-\frac{{{M}_{-}\varphi }_{f}{c}_{-}{D}_{-}}{{\text{RT}}}{\text{grad}}\left({\mu }_{-}\right).$$

Therein ${D}_{+}$ and ${D}_{-}$ are the diffusivities of the cations and anions. In Eqs. (7) and (8), the first term describes the convection of ions with the motion of the fluid, and the second term describes the diffusion due to gradients in the electrochemical potential of the ions. In soft biological tissues, charge accumulations are equilibrated usually within a timescale of few ns (Grodzinsky et al. 1981), thus electroneutrality at every position and every time can be assumed for the present application (Lai et al. 2000). The electroneutrality condition reads

9 $${c}_{+}={c}_{-}+{c}_{{\text{fc}}}\left(J\right),$$

with ${c}_{\text{fc}}$ being the fixed charge density. It changes from its reference values ${c}_{\text{fc}}^{\text{ref}}$ with the deformation of the solid matrix (Ehlers, 2009):

10 $${c}_{{\text{fc}}}=\frac{1-{\varphi }_{\text{s}}^{{\text{ref}}}}{J-{\varphi }_{s}^{{\text{ref}}}}{c}_{{\text{fc}}}^{{\text{ref}}}.$$

The governing equations of the quadriphasic system are given as follows. Assuming quasistatic conditions, no external body forces, incompressibility of each phase, a saturated medium and negligible contribution of the ions to the overall volume, the conservation of momentum and volume read (Ateshian and Weiss 2013; Sun et al. 1999)

11 $${\text{div}}\left[{\varvec{\upsigma}}\right]=0,$$

12 $${\text{div}}\left[{{\varvec{v}}}_{\text{s}}+{{\varvec{j}}}_{\text{f}}\right]=0.$$

The Cauchy stress ${\varvec{\upsigma}}$ of the mixture results from the solid elastic stress ${{\varvec{\upsigma}}}_{s}$**,** which is the sum of the stored strain energy and the hydrostatic pressure contribution and is given by

13 $${\varvec{\sigma}}={{\varvec{\sigma}}}_{\text{s}}-p{\varvec{I}}$$

where ${\varvec{I}}$ being the identity tensor. The continuity equations for the ions read (Ateshian et al. 2013; Sun et al. 1999)

14 $$\frac{{D}^{s}\left(J{\varphi }_{\text{f}}{M}_{+}{c}_{+}\right)}{{\text{Dt}}}+J{\text{d}}{\text{iv}}\left[{{M}_{+}{\varvec{n}}}_{+}\right]=0,$$

15 $$\frac{{D}^{s}\left(J{\varphi }_{\text{f}}{{M}_{-}c}_{-}\right)}{{\text{Dt}}}+J{\text{d}}{\text{iv}}\left[{M}_{-}{{\varvec{n}}}_{-}\right]=0,$$

with $\frac{{D}^{s}}{Dt}$ denoting the material time derivative following the solid (Ateshian and Weiss 2013; Holzapfel 2000). Dividing the mass balance of all charges by their molar mass, multiplying each by the respective charge number and applying the electroneutrality condition yield the electrical current condition:

16 $${\text{div}}\left[{{\varvec{n}}}_{+}\right]-{\text{d}}{\text{iv}}\left[{{\varvec{n}}}_{-}\right]=0.$$

Supplementary Sect. 1.1 describes the finite element implementation of the governing Eqs. (11), (12) and (14–16). Thereby, fluxes are defined according to Eqs. (5), (7) and (8). The only missing element is the stress tensor ${{\varvec{\upsigma}}}_{{\text{s}}}$ in Eq. (13), which is introduced in the next section.

#### Nonlinear dissipative model of the solid

The nonlinear elastic dissipative behavior of the solid matrix is described by a Rubin- and Bodner-type strain energy function ${{\varvec{\Psi}}}_{{\text{s}}}$, (Rubin and Bodner 2002) which is extended from the previous work (Mauri et al. 2016a, b; Sachs et al. 2021).

17 $${\boldsymbol{\Psi }}_{s}={\varphi }_{s}^{{\text{ref}}}\frac{{\mu }_{0}}{2q}\left[{\text{exp}}\left({qg}\right)-1\right].$$

The term $g={g}_{\text{m}}+{g}_{{\text{fe}}}+{g}_{{\text{fd}}}+{g}_{{\text{fs}}}$ includes contributions from matrix and fibers. The terms ${g}_{\text{m}}$ and ${g}_{\text{fe}}$ correspond to elastic matrix and fiber contributions. The terms ${g}_{\text{fd}}$ and ${g}_{\text{fs}}$ are dissipative contributions of the fibers. They are defined as follows:

18 $${g}_{\text{m}}={m}_{1}\left[{\text{tr}}\left({{\varvec{F}}{\varvec{b}}}_{0}{{\varvec{F}}}^{T}\right)-3\right]+\frac{{m}_{1}}{{m}_{2}}\left[{\left(J{J}_{0}\right)}^{-2{m}_{2}}-1\right]$$

19 $${g}_{{\text{fe}}}=\frac{{m}_{{\text{fe}}}}{{m}_{\text{4e}}}\frac{1}{N}\sum_{i=1}^{N}\langle {\lambda }_{0}^{i}{\lambda }_{{\text{fe}}}^{i}-1{\rangle }^{2{m}_{\text{4e}}}$$

20 $${g}_{{\text{fd}}}=\frac{{m}_{{\text{fd}}}}{{m}_{\text{4d}}}\frac{1}{N}\sum_{i=1}^{N}\langle {\lambda }_{{\text{fd}}}^{i}-1{\rangle }^{2{m}_{\text{4d}}}$$

21 $${g}_{{\text{fs}}}=\frac{{m}_{{\text{fs}}}}{{m}_{\text{4s}}}\frac{1}{N}\sum_{i=1}^{N}\langle {\lambda }_{{\text{fs}}}^{i}-1{\rangle }^{2{m}_{\text{4s}}}$$

**Fig. 2:**
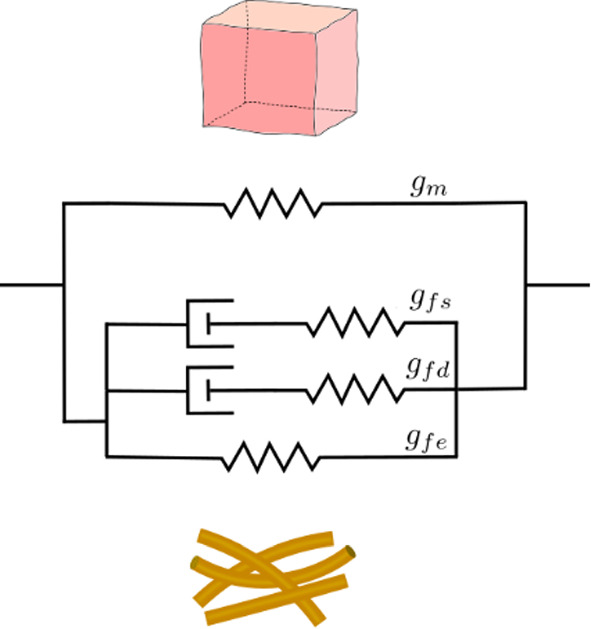
The response of the skin’s solid constituents is illustrated by a spring–damper analogy. The top spring describes the elastic matrix, which is set in parallel with the fibers that have three contributions in parallel (top to bottom): a slow and fast dissipative contribution, and an elastic one

Therein, the material parameters ${\mu }_{0}$ and $q$ influence the overall behavior, ${m}_{1}$ and ${m}_{2}$ determine the response of the matrix and ${m}_{{\text{fe}}}$, ${m}_{4e}$, ${m}_{{\text{fd}}}$, ${m}_{4d}$, ${m}_{{\text{fs}}}$ and ${m}_{4s}$ describe the behavior of the fibers. The initial deformations ${{\varvec{b}}}_{0}$=${{\varvec{F}}}_{0}{{\varvec{F}}}_{0}^{{\varvec{T}}}$, ${J}_{0}$ and ${\lambda }_{0}^{i}$ result from swelling due a nonzero osmotic pressure in the swollen reference condition (Wahlsten et al. 2019). The stretches of the elastic and dissipative fiber contribution are denoted by ${\lambda }_{{\text{fe}}}^{i}$, ${\lambda }_{{\text{fd}}}^{i}$ and ${\lambda }_{{\text{fs}}}^{i}$. The positive scalar $N$ accounts for the number of fiber families. The fiber families are distributed equally within the plane parallel to the skin surface with a small out-of-plane pitch. The Macaulay brackets $\left\langle {-} \right\rangle$ ensure fibers only being active in tension. Figure 2 illustrates the solid contribution of the material model using a simplified spring–damper analogy. The matrix and the fibers have an elastic contribution. Two dissipative elements are included to represent fast and slow dissipative contributions of the fibers.

The stretches ${\lambda }_{{\text{fd}}}^{i}=\Vert {{\varvec{m}}}_{{\text{fd}}}^{i}\Vert$ and ${\lambda }_{fs}^{i}=\Vert {{\varvec{m}}}_{{\text{fs}}}^{i}\Vert$ are defined from evolution equations as follows (Mauri et al. 2016a, b; Wahlsten et al. 2019):

22 $${\dot{{\varvec{m}}}}_{{\text{fd}}}^{i}=\dot{{\varvec{F}}}{{\varvec{F}}}^{-1}{{\varvec{m}}}_{{\text{fd}}}^{i}-{k}_{{\text{fd}}}{\text{tr}}\left({{\varvec{\sigma}}}_{{\text{fd}}}^{i}\right){{\varvec{m}}}_{{\text{fd}}}^{i},$$

23 $${\dot{{\varvec{m}}}}_{{\text{fs}}}^{i}=\dot{{\varvec{F}}}{{\varvec{F}}}^{-1}{{\varvec{m}}}_{{\text{fs}}}^{i}-{k}_{{\text{fs}}}{\text{tr}}\left({{\varvec{\sigma}}}_{{\text{fs}}}^{i}\right){{\varvec{m}}}_{{\text{fs}}}^{i}.$$

With ${{\varvec{m}}}_{{\text{fd}}}^{i}$ and ${{\varvec{m}}}_{{\text{fs}}}^{i}$ describe the current fiber vectors, ${k}_{{\text{fd}}}$ and ${k}_{{\text{fs}}}$ are dissipation rates and ${{\varvec{\upsigma}}}_{{\text{fd}}}^{i}$ and ${{\varvec{\upsigma}}}_{{\text{fs}}}^{i}$ are the Cauchy stresses of the respective fiber contribution.

The following table summarizes all parameters of the quadriphasic model (Table 1):

**Table 1:** Model parameters and their interpretation

${\mu}_{0} \, \left[{\text{MPa}}\right]$	Pre-factor	$\vartheta \, \left[^\circ \right]$	Out-of-plane angle
$q$	Nonlinearity factor	${k}_{\text{fd}} \, \left[{\text{mm}}^{2}{\text{N}}^{-1}{\text{s}}^{-1}\right]$	Dissipation rate
${m}_{1}$	Matrix parameter	${k}_{\text{fs}} \, \left[{\text{mm}}^{2}{\text{N}}^{-1}{\text{s}}^{-1}\right]$	“
${m}_{2}$	“	${k}_{0} \, \left[{\text{m}}^{4}{\text{N}}^{-1}{\text{s}}^{-1}\right]$	Permeability
${m}_{\text{fe}}$	Fiber stiffness	$\kappa$	Nonlinearity parameter of permeability
${m}_{\text{fd}}$	“	${c}_{\text{fc}} \, \left[M\right]$	Fixed charge density
${m}_{\text{fs}}$	“	${{\text{D}}}_{+}\left[{\text{m}}^{2}{\text{s}}^{-1}\right]$	Diffusivity
${m}_{4\text{e}}$	Fiber nonlinearity	${{\text{D}}}_{-}\left[{\text{m}}^{2}{\text{s}}^{-1}\right]$	“
${m}_{4\text{d}}$	“	${\varphi }_{\text{s}}^{\text{ref}}$	Reference solid volume fraction
${m}_{4\text{s}}$	“		

### Experimental methods

The parameters included in the model equations are determined based on a wide range of experimental observations. Data from our previous mechanical experiments on human skin are considered, including time-dependent uniaxial and biaxial tension tests. Among these, relaxation experiments with change of the osmolarity of the bath help determining the parameters associated with fluid and ions potentials. To improve the characterization of the mechanical response and in particular of the fluid and ionic fluxes in the tissue, new displacement-controlled and force-controlled out-of-plane compression experiments were performed, considering also a change of the ionic environment. Compression experiments are relevant in that the contribution of collagen fibers to the stress is negligible, thus providing useful input for the determination of the parameters related to the motion of the other model components. The following section describes the new experiments, while references (Wahlsten et al., Sachs et al.) report the details on the other testing configurations.

#### Compression test: experimental setup

**Fig. 3:**
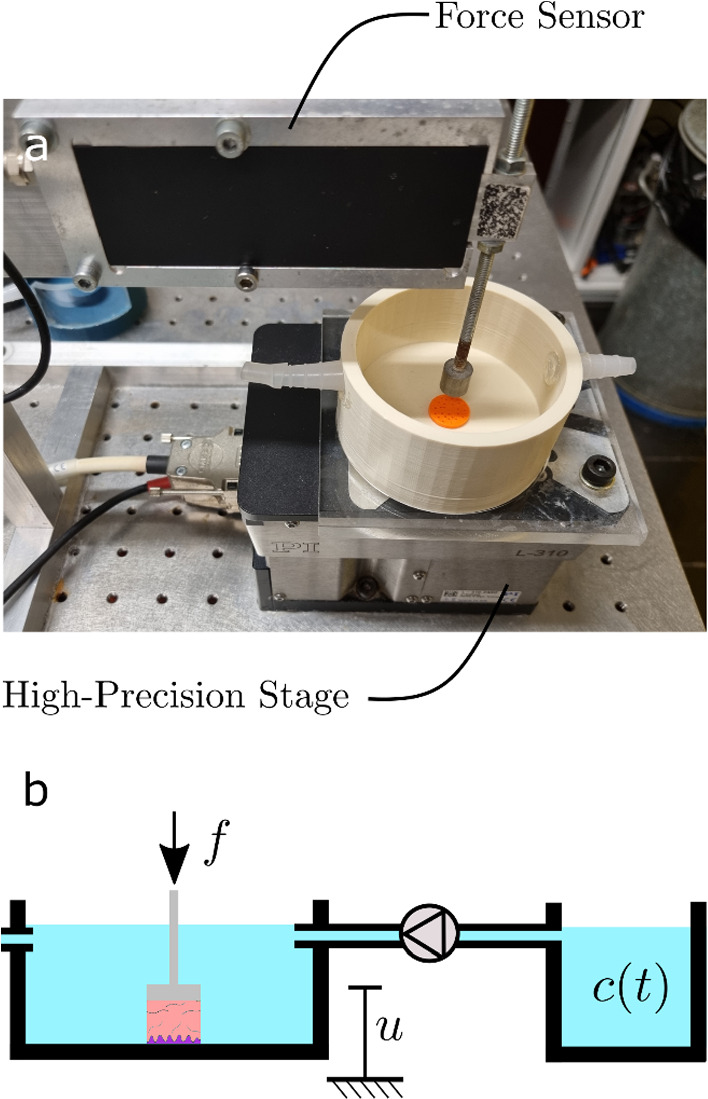
Experimental set-up for unconfined compression experiments. **a** Photograph of the test setup. The custom-built water bath (white) has an inlet and outlet, connected to a large reservoir of external bath (not shown). The orange disk corresponds to the location of the specimen. The high-precision vertical stage below the water bath and the force sensor is fixed on the vertical rod holding the porous filter on top of the specimen. **b** Sketch of the full setup consisting of the testing bath (left) and the reservoir (right, not to scale). The chambers are connected through a pump. During the measurements, both force $$ {{f}}$$ and displacement $$\it {\text{u}}$$ are continuously measured. Further, prescribing the ions concentration $$c(t)$$ in the reservoir allows to control the concentration of the testing bath

Unconfined compression experiments were performed using a custom-built setup, as shown in Fig. 3. The setup consists of a force sensor (2 N, A. S. T.—Angewandte System Technik GmbH Mess—und Regeltechnik, Dresden, Germany), a precision stage for vertical movement (Physik Instrumente (PI) GmbH, Karlsruhe, Germany), a custom-built testing bath, a reservoir and a peristaltic pump (stepper motor: Trinamic Motion Control GmbH, Hamburg, Germany; pump head: Watson Marlow AG, Zürich, Switzerland). The pump connects the testing bath with the reservoir, which is used to control the ionic concentration and has a 10 times bigger volume than the testing bath. The pump ensures that within 5 min, the total volume of the testing bath is exchanged with fluid coming from the reservoir. The whole set-up is controlled via a custom written code in MATLAB (The Mathworks, Natick, MA, USA).

#### Specimen preparation

Human breast and abdominal skin specimens were provided by the University Hospital of Zürich with assistance of the SKINTEGRITY.ch biobank (EK 647 and EK 800). All samples used were surplus material from routine surgeries. Informed consent had been obtained from all patients, and all experiments were conducted according to the principles set out in the WMA Declaration of Helsinki and the Department of Health and Human Services Belmont Report. The use of material for research purposes was approved by the local ethic commission (BASEC ID: 2017–00684). Donors were female between 25 and 40. A total of 11 samples were used, 5 for simple compression experiments and 6 for change of bath experiments. Both breast and abdominal skin specimens were used for each test. The specimens were kept on phosphate-buffered saline solution until testing, which was performed within 18 h after tissue collection.

For testing, the adipose tissue was carefully removed using a surgical scalpel. Specimens were then cut into samples of 15-mm diameter using a circular stamp, which was quickly moved vertically into to sample to ensure a rapid and clean cut. The thickness of all samples was about 2 mm. Before testing, the samples were equilibrated in either saline solution or distilled water for 1 h. The sample was then placed with the epidermis facing down in the water bath, which was kept at room temperature.

#### Testing procedure

For each test, the vertical rod approaches the sample slowly with a vertical motion until a reference force of 1 mN is reached. This defines the reference configuration for the experiment and identifies the initial vertical dimension (thickness) of the specimen. For displacement-controlled compression tests, the samples were then compressed with a vertical displacement rate of 0.001 $\frac{{\text{mm}}}{{\text{s}}}$ until a force of 500 mN was reached. The displacement was then kept constant for 30 min, and the force magnitude was recorded. All displacement-controlled tests were performed in 0.15 M saline solution. For force-controlled compression experiments with change of bath, the force was increased from 1 to 25 mN within 1 min, kept constant thereafter until the end of the experiment and the displacement was recorded. In order to complement the observations in relaxation experiment with change of bath (which always started from 0.15 M NaCl, see (Wahlsten et al. 2019)), the present experiments considered tissue samples equilibrated in deionized water and a change of bath to 0.15 M NaCl solution after 2 h. Postprocessing of the results of all experiments was performed with a custom written code in MATLAB (The Mathworks, Natick, MA, USA).

#### Monotonic uniaxial and relaxation experiments

Parameter determination considered also the experimental data from Wahlsten et al. 2019 where monotonic uniaxial tests and relaxation experiments with change of bath were performed on human skin. Similar as for the new experiments, skin samples from breast and abdomen had been used. The experiments are briefly described here. Both monotonic uniaxial and relaxation experiments were performed on specimens with gauge dimensions of $20 \times 5 \times 2\;\text{m}{{\text{m}}}^{3}$ in a saline bath with a concentration of 0.15 M NaCl. In monotonic uniaxial tests, the specimens were elongated with a nominal strain rate of $0.001 {{\text{ s}}}^{-1}$. Extension and lateral contraction (in-plane and out-of-plane) were determined based on the analysis of images of two cameras. In relaxation experiments, specimen was elongated with a much higher nominal strain rate $\left(0.05 {{\text{ s}}}^{-1}\right)$ until a predefined force of 1 N was reached. Samples were then held at that stretch for 30 min, and force relaxation was recorded. For some of the relaxation experiments, the ionic bath was changed during the experiment. To this end, the bath was emptied after 2 min of relaxation and refilled with either a hypertonic (0.60 M NaCl) or hypotonic (deionized water) solution (for details, see Wahlsten et al. 2019).

## Results

### Experimental observations

**Fig. 4:**
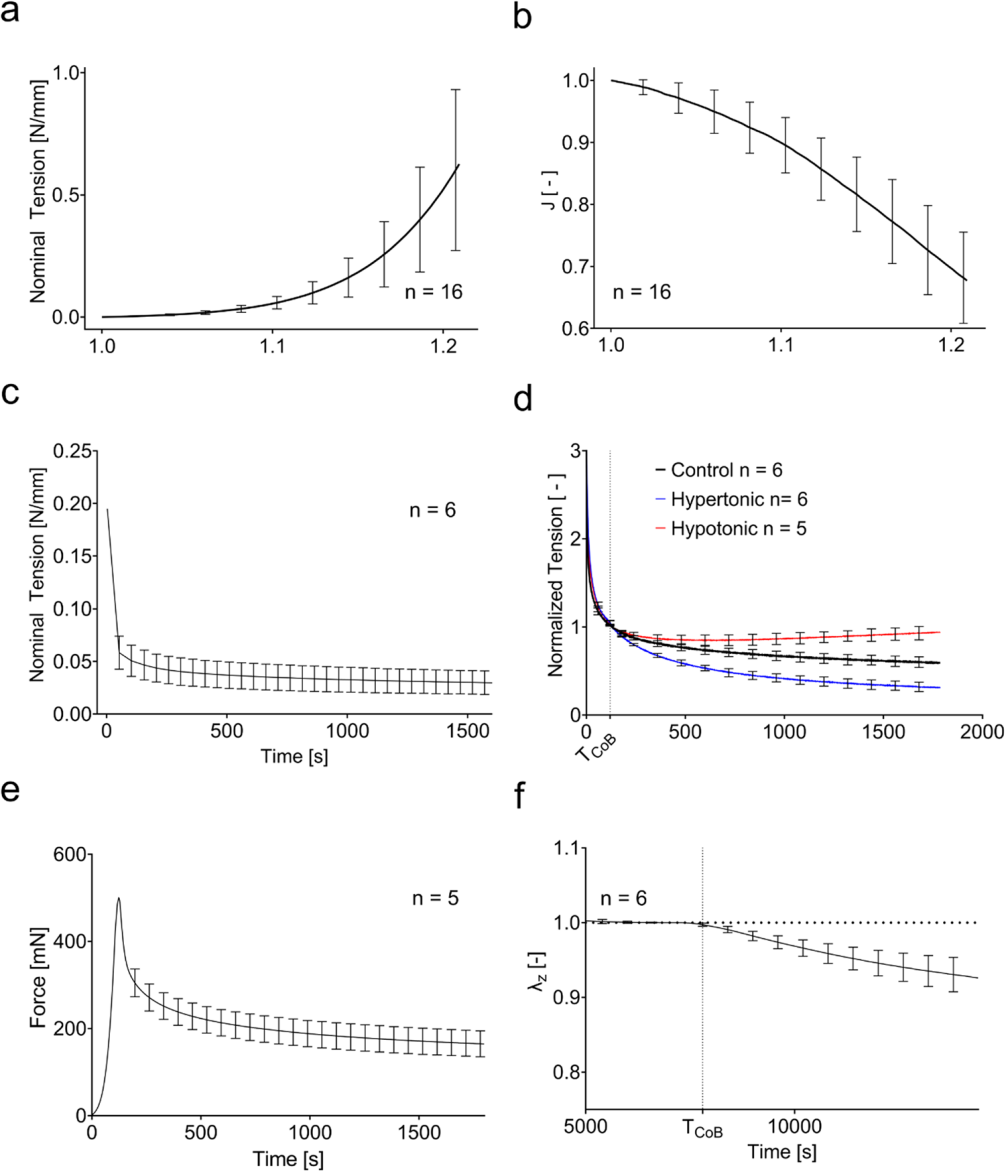
Experimental results of the different mechanical testing procedures. **a** *J*-shaped tension–stretch curves and **b** corresponding reduction of volume in terms of $J={\text{det}}(\mathbf{F})$ in monotonic uniaxial tension. **c** Nominal tension in relaxation. **d** Tension–relaxation normalized with respect to the value at change of bath (*T*_CoB_ = 120 s) for the control (black), for a change to deionized water (hypotonic, red) and a change to 0.6 M saline (hypertonic, blue). **e** Compression force in displacement-controlled unconfined compression experiments. **f** Out-of-plane stretch for a force-controlled unconfined compression with change of bath from deionized water to physiological saline at *T*_CoB_ = 7200 s

The experimental results are summarized in Fig. 4. The monotonic uniaxial tests are represented as nominal membrane tension, i.e., force per unit reference width, as a function of longitudinal stretch. They are characterized by the J-shaped typical for soft collagenous tissues. A pronounced contraction in the lateral and out-of-plane direction leads to about 35% volume decrease for a stretch of 1.2.

Relaxation experiments, shown in Fig. 4c, show a rapid force reduction after the initial loading phase. The force magnitude drops to about ¼ of its peak value within few minutes. The relaxation rate then decreases; however, a negative slope is present until the end of the experiment. The change of bath alters the tension–relaxation response of skin. As shown in Fig. 4d, a change to deionized water increases the tension, while a change to higher salinity decreases it. The magnitude of the tension change is similar in both directions.

In displacement-controlled compression tests, shown in Fig. 4e, skin shows a force reduction after the initial peak associated with tissue compression. Within 30 min, it reduces to less than half of the initial values. Force-controlled compression experiments with change of bath again highlight the coupling of chemical potential and mechanical forces in skin. The change of bath was performed after 2 h to ensure that the skin was in equilibrium. As shown in Fig. 4f, an increase in salinity of the bath leads to a decrease in thickness of the sample. When changing from distilled water to 0.15 M NaCl solution, the material slowly contracts by about 10% in thickness direction. Comparison with Fig. 4d shows that in force-controlled out-of-plane compression experiments, the skin requires more time to adapt to a change of ionic concentration of the external bath when compared to in-plane tension–relaxation tests.

### Inverse analysis

This section presents the results of the iterative optimization procedure for determining the model parameters of the dermis. The initial parameters were taken from our previous study, which also included in vivo suction measurements (Sachs et al. 2021). Even if not presenting the in vivo results in this contribution, agreement with suction data was verified for the final set of parameters of the present model. This is important as suction induces mainly an equibiaxial state of tension of skin, thus providing a confirmation of the validity of the model prediction for a multiaxial deformation state.

The diffusivities of the ions ${({\text{D}}}_{-},{{\text{D}}}_{+})$, the solid volume fraction in the reference configuration $({\varphi }_{\text{s}}^{{\text{ref}}})$ and the deformation dependency of the permeability $(\kappa )$ were chosen according to values available in the literature (Nakagawa et al. 2010; Wahlsten et al. 2019; Zhang et al. 2009).

Computational models for all experiments were performed in COMSOL Multiphysics 6.1, COMSOL AB, Stockholm, Sweden. More details of the finite element simulations are shown in the supplementary material (Sects. 1.2 and 1.3). A gradient-based iterative optimization procedure was applied using the *MATLAB Optimization Toolbox* and the *MATLAB LiveLink to COMSOL*. The material parameters obtained from the optimization run are summarized in Table 2.

**Table 2:** Material parameters of the model corresponding Eqs. (1)–(23)

${\mu }_{0} \, \left[{\text{MPa}}\right]$	$0.01\times {10}^{-2}$	$\vartheta \, \left[^\circ \right]$	$18$
$q$	$0.6$	${k}_{\text{fd}} \, \left[{\text{mm}}^{2}{\text{N}}^{-1}{\text{s}}^{-1}\right]$	$1\times {10}^{-2}$
${m}_{1}$	$0.2$	${k}_{\text{fs}} \, \left[{\text{mm}}^{2}{\text{N}}^{-1}{\text{s}}^{-1}\right]$	$0.1\times {10}^{-2}$
${m}_{2}$	$1$	${k}_{0} \, \left[{\text{m}}^{4}{\text{N}}^{-1}{\text{s}}^{-1}\right]$	$0.5\times {10}^{-13}$
${m}_{\text{fe}}$	$150$	$\kappa$	$2$
${m}_{\text{fd}}$	$600000$	${c}_{\text{fc}} \, \left[M\right]$	$3.5\times {10}^{-2}$
${m}_{\text{fs}}$	$4000000$	${{\text{D}}}_{+}\left[{\text{m}}^{2}{\text{s}}^{-1}\right]$	$1 \times {10}^{-9}$
${m}_{\text{4e}}$	$1.34$	${{\text{D}}}_{-}\left[{\text{m}}^{2}{\text{s}}^{-1}\right]$	$1 \times {10}^{-9}$
${m}_{\text{4d}}$	$3.6$	${\varphi }_{\text{s}}^{{\text{ref}}}$	$0.3$
${m}_{\text{4s}}$	$3.2$		

The correspondence between measurements and model prediction is shown in the following sections. Note that a modification of the representation of the osmotic pressure had to be introduced for very dilute solutions, which deviates from the ideal behavior. This modification is described in Sect. 3.2.4.

#### Monotonic uniaxial tension

**Fig. 5:**
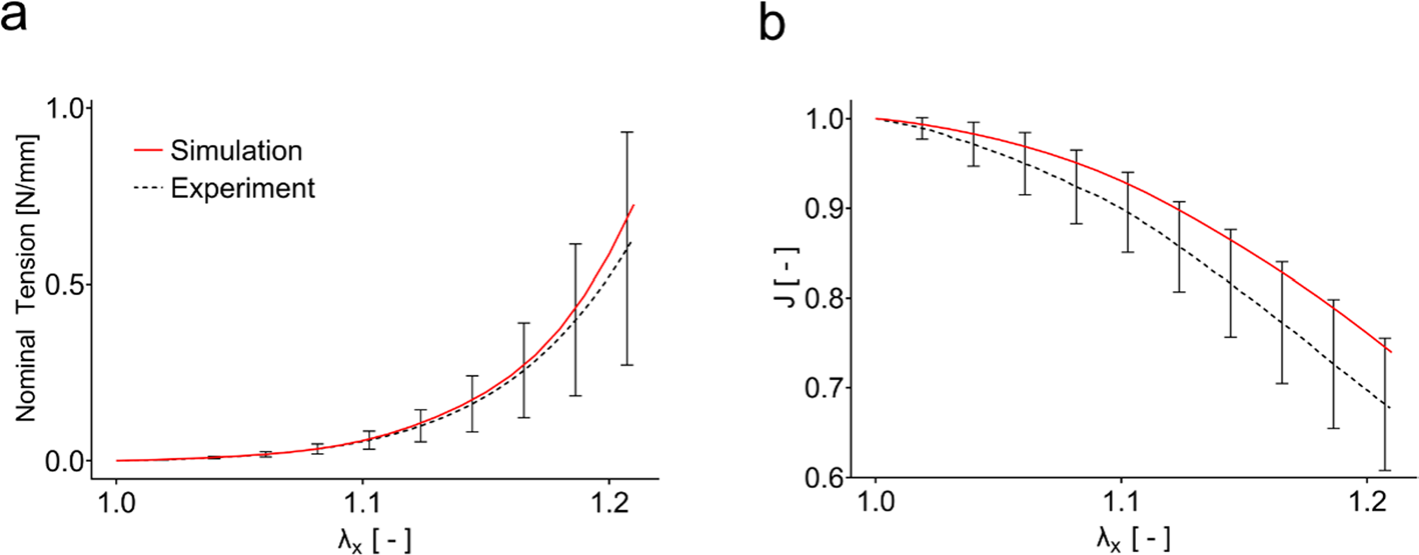
Inverse analysis of the uniaxial ex vivo experiment. The computational model (red, solid) captures **a** the *J*-shaped stress strain curve as well as **b** the volume reduction observed in the experiments (black, dashed)

Figure 5 shows the results of the quadriphasic model to the monotonic uniaxial experiments. The model reproduces to a good extent the mechanical and kinematic behavior of the material. The nonlinear stress–strain relationship mainly results from the nonlinear strain energy function of the fibers, which provide the main contribution to the deformation energy and are also responsible for the reduction in volume. In fact, reorientation of fibers upon loading leads to a decrease in width and thickness resulting in fluid outflux, which reduces the volume of the specimen (cf. (Ehret et al. 2017)).

#### Relaxation with change of bath

**Fig. 6:**
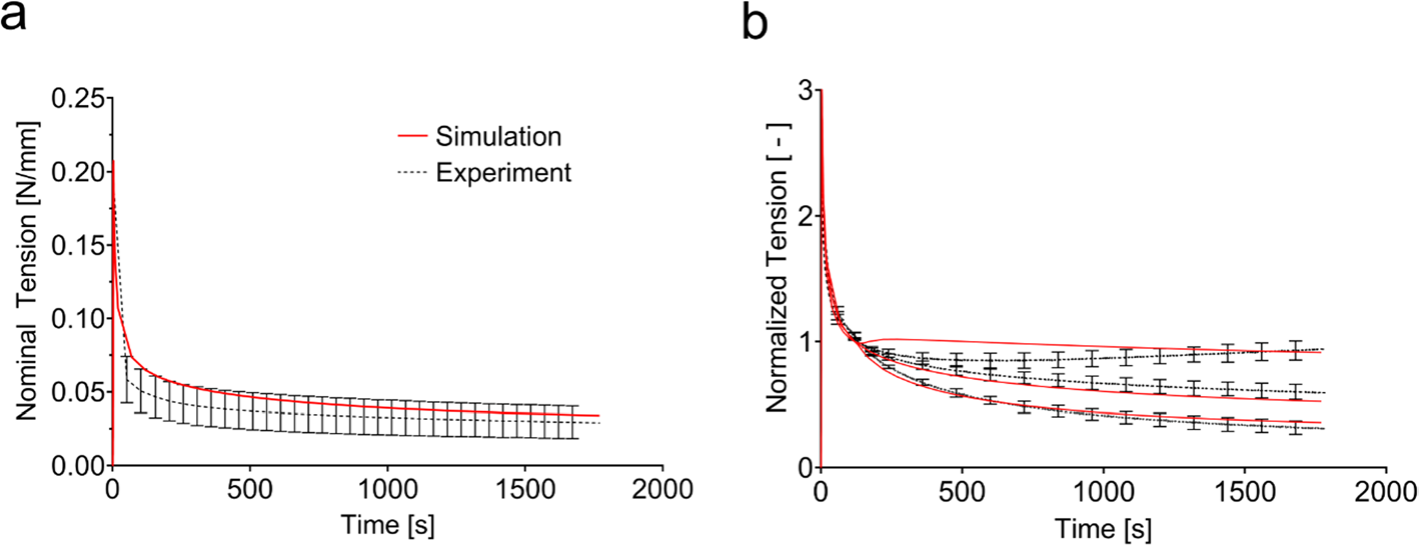
**a** The model formulation with two dissipative components allows fitting both the first fast and the following slow relaxation phase. **b** The magnitude of the change of force when changing the ionic environment is well represented by the model. When changing to deionized water, the model (red lines) predicts a faster initial response compared to the experimental observation (solid lines)

Previously performed relaxation tests are also reasonably well captured by the model. As shown in Fig. 6a, not only the first strong relaxation within the first 2 min, but also the long-lasting weak relaxation is represented by the model. In relaxation experiments with change of bath, the quadriphasic model captures the magnitude of the force variation, as shown in Fig. 6b. The permeability of the tissue thereby governs the timescale of the tissue to adapt to a change in the ionic concentration of the bath, with a lower permeability leading to a slower adaption of the force. While the current model predicts well the temporal evolution to a change in higher salinity, it initially overestimates the change in force after changing to deionized water. As mentioned above, the osmotic contribution was modified for this case, see Sect. 3.2.4.

#### Compression with change of bath

**Fig. 7:**
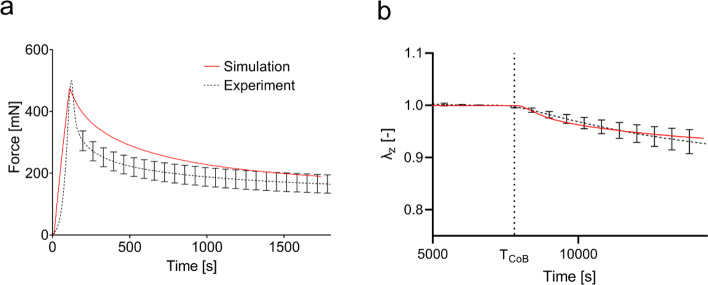
**a** In displacement-controlled compression experiments, the calculated force relaxation agrees to a good extent with the experiments. **b** The simulation of a change of bath from deionized water to 0.15 M saline overestimates the rate of contraction in the beginning but captures the magnitude of the change at the end of the experiment

The comparison of the quadriphasic model predictions with data of the newly performed compression experiments is shown in Fig. 7. The model reproduces the relaxation in displacement-controlled compression tests (Fig. 7a). The strong reduction of the measured force results from fluid efflux from the tissue. Once again, the permeability strongly influences the temporal characteristics of this behavior. Simulation of compression experiments with change of bath reproduces the observed vertical contraction of the tissue due to fluid efflux caused by an increase in external salinity, as shown in Fig. 7b. Like during a change of the bath to deionized water in the relaxation experiments presented in Fig. 6b, the model overestimates the response in the first minutes after the changes of bath. In the long-term, however, simulation and experimental data agree to a good extent. Also, for this case, an adaption of the osmotic contribution was applied as described in Sect. 3.2.4.

#### Human skin exhibits non-ideal osmotic pressure for very dilute solutions

The limit case of zero ions in the immersion bath cannot be considered by the model. In fact, free ions are needed in order to satisfy the electroneutrality condition, otherwise leading to an unbound magnitude of the electrical potential. When the concentration of ions in the bath is strongly reduced, the behavior deviates from the assumptions considered in Donnan’s equilibrium (ideal case). In fact, recent experimental observations on cartilage showed that the ideal Donnan equilibrium overestimates the osmotic pressure for very dilute solutions (Zimmerman et al. 2021). Zimmerman et al. incorporated the non-ideal osmotic pressure by introducing an “effective” fixed charge density,” which reduces the “true” fixed charge density for very dilute solutions, in line with the approach proposed by Franz 2014. The previous studies explained this behavior by the process of “counterions condensation.” Thereby, it is proposed that cations “condense” on the charged chains and by that lower their effective negative charge (Frank et al. 1990; Manning 1969). The theory was subsequently revisited and extended (Bret and Zimm 1984; Deserno and Holm 2001).

**Fig. 8:**
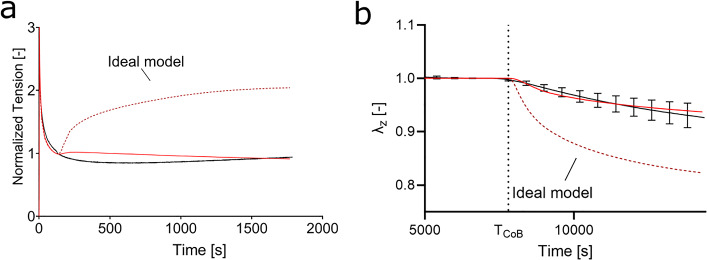
The osmotic pressure predicted based on a change to distilled water (red, dotted in a and b) overestimates the change of force in relaxation experiments **a** and the change in tissue thickness. **b** In both cases, the experimental results (black) are better represented with a correction to the osmotic contribution for very dilute solutions (red, solid)

Our results are in line with the previous experimental findings. In fact, following an ideal formulation of the osmotic pressure, the model captures the observed change in force from a physiological saline to a hypertonic solution, shown in Fig. 6b. However, it would strongly overestimate the electrochemical effects when changing from 0.15 M saline to deionized water (hypotonic solution) or vice versa, as shown in Fig. 8a and b. The strong change in forces results from the excessive increase in osmotic pressure predicted by the ideal Donnan equilibrium for distilled water. Thus, when calculating the change of bath osmolarity to deionized water, calculations showed that by considering conditions of an external ion concentration of 0.06 M as boundary condition, acceptable agreement could be obtained with the measurements. This correction is in line with the adaption of osmotic pressure proposed in Zimmerman et al. 2021.

## Discussion

### The permeability as a key parameter

The inverse analysis for all considered configurations showed a reasonable representation of the force–deformation characteristics and their temporal evolution. While further improvement of the fits of the average curves may be possible (for instance through dedicated optimization algorithms), the large scatter of the experimental data indicates that specific model parameters may be needed for each donor and body location. In fact, the observed scatter can probably be attributed to specific microstructural features of the different samples, but the number of samples available was not sufficient to quantify differences in mechanical response between abdominal and breast skin of the different patients.

An important feature of the new model is the consideration of a long-term dissipative term associated with the fibers. This modification is important for the representation of the relaxation response for times longer than few minutes. For both tension–relaxation as well as compression experiments, the analysis demonstrated that the permeability is an important parameter governing the timescale of the response of the tissue to changes in both the mechanical and the chemical environment. A summary of previous research on various tissues provided by Swartz and Fleury (2007) indicates a wide range of tissue permeability with orders of magnitude between ${10}^{-15}$ and${10}^{-12}\frac{{\text{m}}^{4}}{{\text{Ns}}}$. The difference of three orders of magnitude results in the time scale for equilibrium being in the range of few seconds to several minutes. The best fit over all experiments was obtained for a permeability value of ${k}_{0}={5 \times 10}^{-14}\frac{{\text{m}}^{4}}{{\text{Ns}}}$. We note that this referential value further decreases when the tissue volume reduces during deformation according to Eq. (6). For example, with the current parameters, the volume change of $J=0.7$ reported in Fig. 4b would reduce the effective permeability to 1/3 of its reference value. While we cannot compare the model permeability with data on human skin, studies on rat dermis (Bert and Reed 1995) and mouse tail skins (Swartz et al. 1999) report permeabilities of $(2\; \text{to}\; 6)\times {10}^{-15}\frac{{\text{m}}^{4}}{{\text{Ns}}}$ and $(5\;\text{to}\; 11)\times {10}^{-13}\frac{{\text{m}}^{4}}{Ns}$, respectively, and thus suggest a wide range for skin tissues in these rodents. Interestingly for purely mechanical tests (Figs. 5, 6a and 7a), a better representation would be possible with a higher permeability. Especially the decrease in force in compression experiments would be captured better. In contrast with that, the temporal response of the change of bath models is better represented with a lower permeability. Here, improved fits could be obtained with a permeability in the order of ${10}^{-15}\frac{{\text{m}}^{4}}{{\text{Ns}}}$.

### The strain-generated electrical potential of the dermis

As shown in studies on cartilage, the deformation of a biological tissue can lead to an electrical potential (Bassett and Pawluk 1972; Grodzinsky et al. 1978). This so-called strain-generated electrical potential is induced by tissue compression, leading to spatial variations of the density of fixed charges. In skin, variations of ion distribution occur also due to in-plane stretch, which is associated with volume reduction and fluid outflow. The former is typically nonhomogeneous thus resulting in a gradient of fixed charges, leading to a “diffusion potential” (Lai et al. 2000). Further, water flowing out of the tissue drags ions with it and creates a current, causing the so-called “streaming potential” (Lai et al. 2000). Both potentials contribute to the strain-generated electrical potential in skin.

**Fig. 9:**
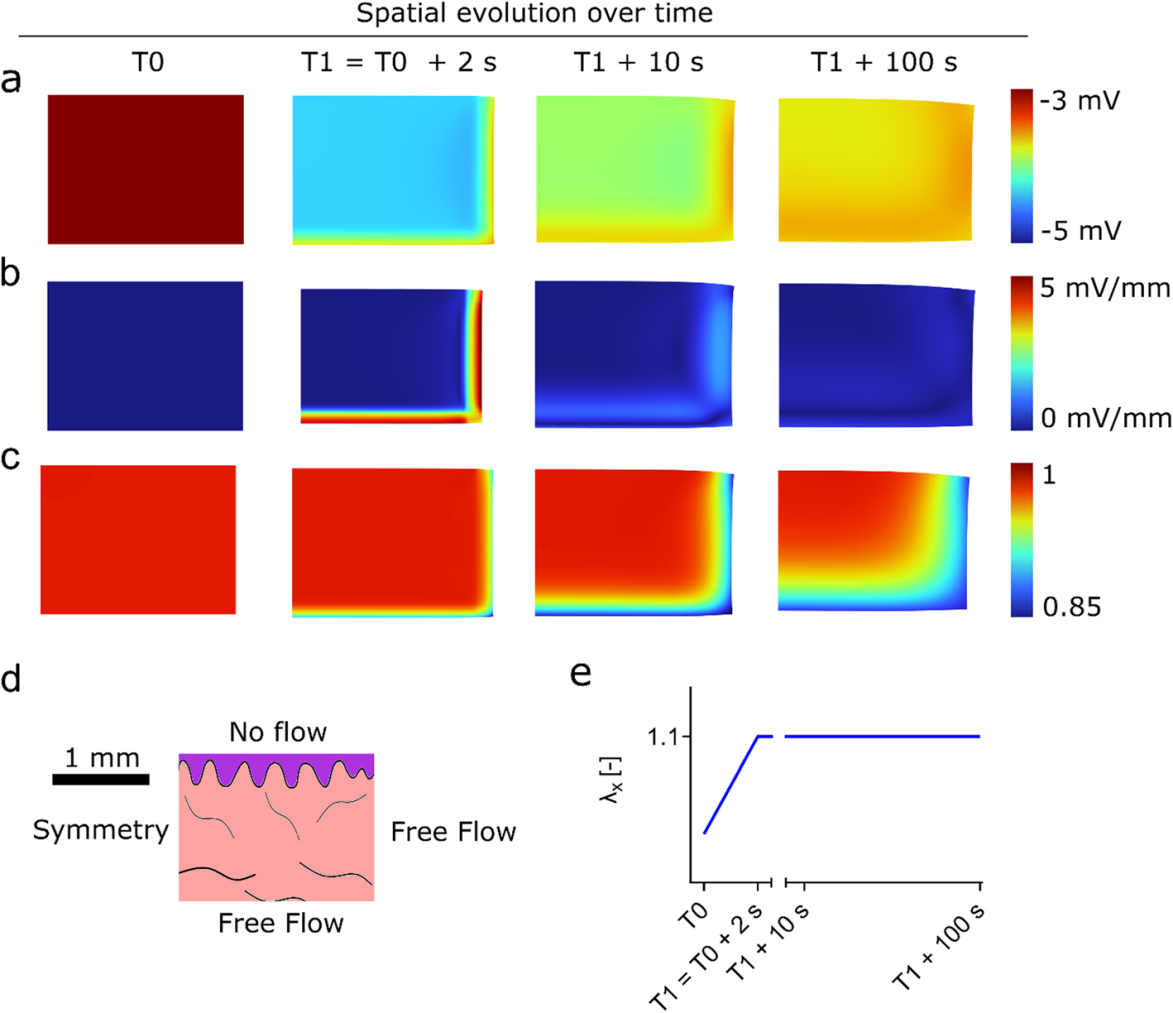
Deformation-induced spatial variations of the electrical potential in human skin. The right half of a cross-section perpendicular to the loading direction is shown for a uniaxial tension of 10% strain within 2 s. **a** Electrical potential inside the tissue. **b** Gradient of electrical potential. **c** Volume change of the tissue during uniaxial relaxation **d** dimensions, orientation of specimen and boundary conditions of fluid and ions and **e** protocol of the relaxation simulation

Figure 9 depicts the results of calculations of the electrical potential (first row), the magnitude of the gradient of the electrical potential (second row) and the volume change (third row) during a uniaxial relaxation test. In the simulation, the specimen was elongated from rest (T0) by 10% within 2 s (T1) and afterwards held at constant displacement. Figure 9 shows half of a cross-section perpendicular to the direction of stretch. The left side marks a symmetry condition and is closed to flows of fluid or ions. A no-flow assumption was also imposed for the top boundary (epidermis). The right and the bottom boundary were free to move, on both boundaries, the chemical potential of the fluid and the electrochemical potential of the ions were prescribed, which were both constants for the duration of the simulation. The results of the simulation are plotted at the beginning of loading, at the end of loading, 10 s and 100 s after the end of the loading. The electrical potential rises quickly inside the tissue from − 3 to − 3.6 mV. Initially, a large gradient exists close to the boundary with magnitudes of up to 15 mV/mm. The gradient of electrical potential then smoothens as the tension force is relaxing. Yet, gradients in chemical potential still remain 100 s after the deformation. A comparison of the electrical potential and the volume decrease, shown in Fig. 9c, underlines the relationship between a change in fixed charge density, resulting from a volume change, and variation of electrical potential. Thereby, volume reduction of around 10% results in changes of electrical potential of several mV. The magnitude of the electrical potential and its gradient depend on the permeability. A parameter study of the influence is provided in Supplementary Fig. 3. The obtained values are lower than experimental values reported in the literature for cartilage, which reach up to 50 mV for fast compression (Grodzinsky et al. 1978). Gradients of electrical potential to induce cell migration in vitro are usually in the order of 50—100 mV/mm (Guo et al. 2010; Lai et al. 2000; Rouabhia et al. 2013). However, lower values of electrical stimulation also showed biological effects. Electric fields of 20 mV/mm promoted cell adhesion, viability and growth in diabetic human skin fibroblasts (Abedin-Do et al. 2021), and values as low as 1 – 10 mV/mm were sufficient to induce cell migration in embryonic fibroblasts (Erickson and Nuccitelli 1984). Deformation-induced gradients of electrical potential in the dermis thus are in the order of magnitude at which one would expect cells to react.

### Model limitations

The new quadriphasic model for the dermal layer of the skin represents an improvement with respect to existing model formulations, yet it includes several simplifying assumptions.

The model neglects spatial variations of properties within the dermis. In the previous work, we have shown through in vivo suction experiments that there are differences in mechanical properties between the papillary and the reticular dermis (Sachs et al. 2021). These differences will also affect the permeability and the fixed charge density, influencing the magnitude and timescale of deformation-induced cues in each layer, such as the electrical potential, osmotic pressure and interstitial fluid velocity. Additionally, our model assumes an in-plane isotropy of skin. Uniaxial experiment in direction of or perpendicular to the Langer lines showed that skin exhibits a pronounced in-plane anisotropy (Ní Annaidh et al. 2012). Consideration of anisotropy is highly relevant for specific applications, for instance when developing location-specific skin models. However, the general findings of the present work are independent of a potential higher extent of skin anisotropy.

As noted above, the permeability has a significant influence on the mechanical response of the dermis and on its temporal evolution following changes in the osmotic environment. Due to its direct link to the interstitial fluid velocity via Darcy’s law, a reliable characterization of the permeability and its dependence on deformation is also necessary to accurately estimate flow velocities in the tissue. The permeability in fibrous tissues is likely to be anisotropic and inhomogeneous, which is neglected in the present formulation (Ateshian and Weiss 2010; Federico and Herzog 2008). As a further simplification, our model neglects the interfibrillar compartment of the collagen fibers. Early studies on the influence of the chemical environment on the mechanical behavior of cartilage point at the need to distinguish the interstitial fluid compartment and the interfibrillar fluid compartment for phenomena involving fluid and ion flows (Katz et al. 1986; Maroudas et al. 1991). Correspondingly, Loret et al. proposed a three-phase model distinguishing between solid phase, interfibrillar fluid and interstitial fluid and showed that the presence of the interfibrillar phase has the capability of damping the temporal evolution of the response to a change of osmolarity of the external bath (Loix et al. 2008; Loret and Simões 2004). Extending our model to include an interfibrillar compartment might rationalize the observed transient mechanical response in case of change of bath experiments.

Furthermore, while our study assesses the changes in mechanical properties on the tissue level, we neglect the heterogeneous microstructure of skin. By assuming an affine deformation, non-affine motion of the collagen fibers also affecting the local volume changes and electrical potential are not taken into consideration (Stracuzzi et al. 2022). Discrete fiber network models were proposed (Eichinger, Grill, et al., 2021; Eichinger et al. 2021a, b; Mauri et al. 2016a, b) that represent collagen as discrete connectors and can provide local information on the distribution of interstitial fluid and fixed charges. Such models could be extended to include the ionic phases as proposed in the present work. Moreover, models explicitly accounting for the presence of cells have been published recently (Eichinger, Grill, et al., 2021; Eichinger et al. 2021a, b). Such discrete or hybrid continuum-discrete models are relevant when studying multiphase mechanotransduction at cellular or sub-cellular length scale. Fitting of these models, however, also requires experimental methods, which help characterizing model parameters for each relevant length scale.

Finally, the current contribution presents a model for the ex vivo behavior of human skin. In the future work, the model should be extended to also represent in vivo conditions. In vivo skin is in a state of “natural tension” (Diridollou et al. 1998; Jacquet et al. 2008) and constantly exchanges fluid, which leaks from the blood capillaries into the interstitial space and is absorbed by the lymphatic capillaries (Swartz and Fleury 2007). This results in specific displacement boundary conditions, but also those for the chemical potential of the fluid and the electrochemical potential of the ions and requires an adaption of the governing equation to include the continuous fluid exchange. Further, the system could be extended to include multiple ionic species (Ateshian et al. 2013).

## Conclusions

Understanding the magnitude and temporal evolutions of electrochemical cues associated with mechanical stimulation of skin is relevant for applications in medicine, for instance when treating chronic wounds, hypertrophic scars or when optimizing processes and products in skin tissue engineering. Computational models thereby allow investigating the complex and nonlinear interaction of electrochemical and mechanical processes in soft tissues. In this study, we introduced a quadriphasic model of the human dermis, representing changes in chemical potential of the fluid and electrochemical potentials of the ions associated with in-plane skin stretch. We performed compression experiments to further characterize the coupling of mechanical and electrochemical effects in the tissue. The experiments highlighted the time-dependent mechanical behavior of human dermis and its dependence on the chemical potential of the fluid environment. Through an inverse analysis, the quadriphasic model was fitted to all experiments, and the results identified the tissue permeability as a key parameter strongly influencing the mechanical, chemical and electrical transient behavior of the dermis. Moreover, comparison with experiments indicates that the osmotic pressure within the dermis is well described by the use of Donnan’s equilibrium for higher concentrations of ions only, including the physiological range. Very dilute solutions, however, require special considerations. Importantly, the new model allowed quantifying deformation-generated gradients of electrical potentials inside skin, and corresponding values associated with in-plane skin deformation are expected to induce a biological response of resident cells. Altogether, the new model, enriched by an additional stress relaxation mechanism and electrochemical coupling, improves our understanding of the mechanome of the human dermis and its mechanical behavior at tissue and cell length scales.

## Supplementary Information

Below is the link to the electronic supplementary material.

Supplementary file1 (DOCX 899 KB)

## Data Availability

Not applicable.
