# Supplementary material for: A quadriphasic mechanical model of the human dermis
Source: Biomech Model Mechanobiol. 2024 Mar 15;23(4):1121–36. doi: 10.1007/s10237-024-01827-5 (PMC11584490; doi:10.1007/s10237-024-01827-5)
Supplement: Supplementary file 1 — Supplementary file1 (DOCX 899 KB) [file 10237_2024_1827_MOESM1_ESM.docx]

Supplementary Material

to “A quadriphasic mechanical model of the human dermis”

David Sachs^1^, Raphael Jakob^1^, Gaetana Restivo^2^, Jürg Hafner^2^, Nicole Lindenblatt^3^, Alexander E. Ehret^1,4^, Edoardo Mazza^1,4^

^1^ ETH Zürich, Institute for Mechanical Systems, Zürich, Switzerland

^2^ Department of Dermatology, University Hospital Zürich, Zürich, Switzerland

^3^ Department of Plastic Surgery and Hand Surgery, University Hospital Zürich, Zürich, Switzerland

^4^ Empa, Swiss Federal Laboratories for Materials Science and Technology, Experimental Continuum Mechanics, Dübendorf, Switzerland

# 1. Finite element implementation

## 1.1 Implementation of the model

The governing equations of the quadriphasic model are implemented using existing sets of equations published by Sun et al. and Ateshian et al. (Ateshian et al., 2013; Ateshian & Weiss, 2013; Sun et al., 1999).

The governing variables are the solid displacement $\boldsymbol{u}$, the chemical potential of the fluid ($\epsilon_{f}$) and the adapted electrochemical potentials of the ions ($\epsilon_{+},\epsilon_{-}$), which are given by (Sun et al., 1999, Eqs. (24) - (26)))

|  | $\epsilon_{f}= \rho_{f}^{T}\left( \mu_{f}-\mu_{f,0} \right),$ | (A 1) |
| --- | --- | --- |
|  | $\epsilon_{+}=exp\left[ \frac{M_{+}}{RT}\left( \mu_{+}-\mu_{+,0} \right) \right],$ | (A 2) |
|  | $\epsilon_{-}=exp \left[ \frac{M_{-}}{RT}\left( \mu_{-}-\mu_{-,0} \right) \right].$ | (A 3) |

They depend on the chemical potential of the fluid $\mu_{f}$ and the electrochemical potentials of the ions $\mu_{+}$ and $\mu_{-}$ given in equations (1), (2) and (3) of the main paper. The flows of fluid ($\boldsymbol{j}_{f}$) and ions ($\boldsymbol{n}_{\boldsymbol{+}}\boldsymbol{,}\boldsymbol{n}_{\boldsymbol{-}}$) relative to the solid can be expressed depending on the adapted potentials as (Eqs. (30) - (31) in (Sun et al., 1999))

|  | $\boldsymbol{j}_{f}=-k\left( J \right)\mathrm{grad}\left( \epsilon_{f} \right)+\frac{RT{k\left( J \right)c}_{+}}{\epsilon_{+}}\mathrm{grad}\left( \epsilon_{+} \right)+\frac{RT{k\left( J \right)c}_{-}}{\epsilon_{-}}\mathrm{grad}\left( \epsilon_{-} \right),$ | (A 4) |
| --- | --- | --- |
|  | $\boldsymbol{n}_{\mathbf{+}}=c_{+}k\left( J \right)\mathrm{grad}\left( \epsilon_{f} \right)-\left[ \frac{\varphi_{f}c_{+}D_{+}}{\epsilon_{+}}+\frac{RTk\left( J \right)c_{+}^{2}}{\epsilon_{+}} \right] \mathrm{grad}\left( \epsilon_{+} \right)-\frac{RTk\left( J \right)c_{+}c_{-}}{\epsilon_{-}}\mathrm{grad}\left( \epsilon_{-} \right),$ | (A 5) |
|  | $\boldsymbol{n}_{\mathbf{-}}=c_{-}k\left( J \right)\mathrm{grad}\left( \epsilon_{f} \right)-\frac{RTk\left( J \right)c_{+}c_{-}}{\epsilon_{+}}\mathrm{grad}\left( \epsilon_{+} \right)-\left[ \frac{\varphi_{f}c_{-}D_{-}}{\epsilon_{-}}+\frac{RTk\left( J \right)c_{-}^{2}}{\epsilon_{-}} \right] \mathrm{grad}\left( \epsilon_{-} \right).$ | (A 6) |

The governing equations of the mixture are (Eqs (37), (38), (41) and (42) of (Sun et al., 1999)): the *balance of* *momentum for the mixture*

|  | $div(\boldsymbol{\sigma})=0,$ | (A 7) |
| --- | --- | --- |

its continuity equation

|  | $\mathrm{div}\left( \boldsymbol{v}_{s} \right)+div(\boldsymbol{j}_{f})=0,$ | (A 8) |
| --- | --- | --- |

the electric current condition

|  | $\mathrm{div}\left( \boldsymbol{n}_{+} \right)-div(\boldsymbol{n}_{-})=0$ | (A 9) |
| --- | --- | --- |

and the ion diffusion-convection equation

|  | $\frac{1}{J}\frac{D^{s}}{Dt}\left( J\varphi_{f}c_{k} \right)+div\left( \boldsymbol{n}_{+} \right)+div(\boldsymbol{n}_{-})=0$ | (A 9) |
| --- | --- | --- |

with $c_{k}=c_{+}+c_{-}$ and $\frac{D^{s}}{Dt}$ represents the material time derivative following the solid (Ateshian et al., 2013; Holzapfel, 2000). Finally, the concentration of the ions result in (Eqs (87) and (88) in (Sun et al., 1999))

|  | $c_{+}=\frac{c_{fc}+\sqrt{c_{fc}^{2}+4\epsilon_{+}\epsilon_{-}}}{2}$ | (A 10) |
| --- | --- | --- |
|  | $c_{-}=\frac{{-c}_{fc}+\sqrt{c_{fc}^{2}+4\epsilon_{+}\epsilon_{-}}}{2}$ | (A 11) |

and the electrical potential is (Eq (94) in (Sun et al., 1999))

|  | $\Psi=\frac{RT}{2F}\ln\left( \frac{c_{-}\epsilon_{+}}{c_{+}\epsilon_{-}} \right).$ | (A 12) |
| --- | --- | --- |

The hydrostatic pressure $p$ results from the definition of the chemical potential of the fluid with

|  | $p= \epsilon_{f}+RT\left( c_{+}+c_{-} \right).$ | (A 13) |
| --- | --- | --- |

Finally, the Cauchy stress of the mixture $\boldsymbol{\sigma}$ is given by (see e.g. (Ehlers et al., 2009))

|  | $\boldsymbol{\sigma}=\boldsymbol{\sigma}_{s}-p\boldsymbol{I}$ | (A 14) |
| --- | --- | --- |

with $\boldsymbol{\sigma}_{s}$ being the solid stress resulting from a Rubin-Bodner type strain energy density function given in equations (17) – (21) and existing literature (Sachs et al., 2021; Wahlsten et al., 2019).

The equations are implemented in COMSOL Multiphysics (COMSOL Multiphysics 6.1, COMSOL AB, Stockholm, Sweden). The momentum equation of the mixture was modeled using the *Structural Mechanics Module* and the *Nonlinear Structural Materials Module*.

The continuity equation of the mixture, the electric current condition and the ion diffusion-convection equation are implemented using the Coefficient Form PDE interface of COMSOL.

## 1.2 Compression simulations


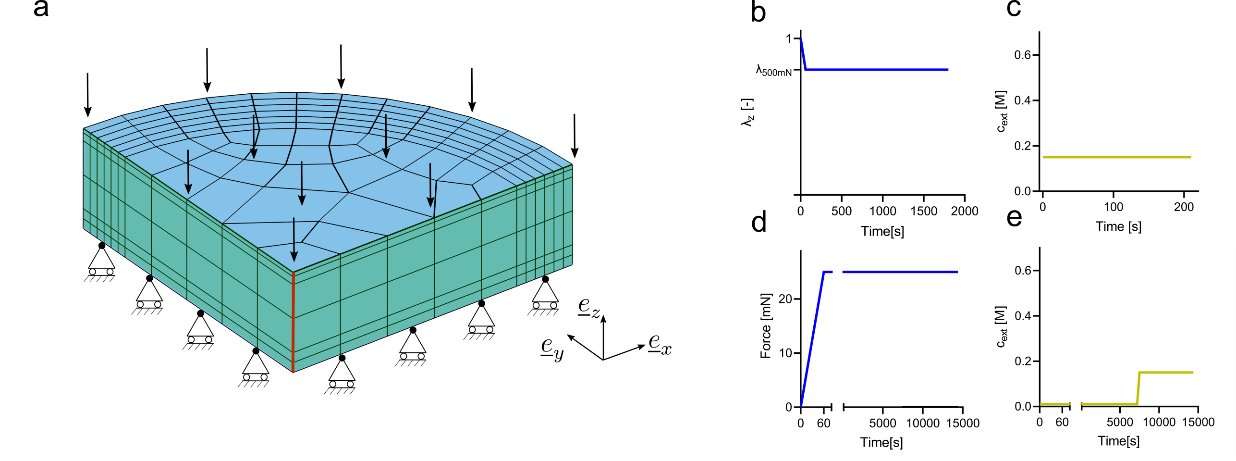


Supplementary Figure 1: (a) Finite element model of the compression simulation. Boundary conditions on (b) displacement and (c) external bath concentration for compression experiment. Boundary conditions on (d) force and (e) external bath concentration for compression with change of bath.

The displacement- and force-controlled compression experiments with change of bath osmolarity are simulated with the finite element model shown in Supplementary Figure 1. One quarter of the domain is simulated. Symmetry conditions are applied on the left and right faces (dark green) for all degrees of freedom. The bottom face is constrained in z-direction. The load related boundary condition, force or displacement, is applied to the top surface (arrows). No friction is assumed between the porous filter, which is not modeled explicitly, and the tissue, while neither fluid nor ions can enter the domain through the bottom boundary. All other boundaries are open to flows of fluid and ions. The elements selected implement quadratic interpolation for displacement of the solid matrix and linear interpolation functions for the chemical potential of the fluid and the electrochemical potentials of the ions. Note that an axisymmetric model could be used for the present simulations with significant reduction of computational costs. The present choice of a 3D model representation is due to possible extensions of the computational work to consider in-plane anisotropy of the skin.

During a change of bath osmolarity the chemical potential of the fluid and the electrochemical potential of the ions is changed at the boundaries within 5 minutes. All other boundary conditions remain unchanged. The stretch was calculated from the total change of thickness of the sample which was evaluated at the center line of the specimen, shown in red in Supplementary Figure 1.

## 1.3 Uniaxial tension simulations


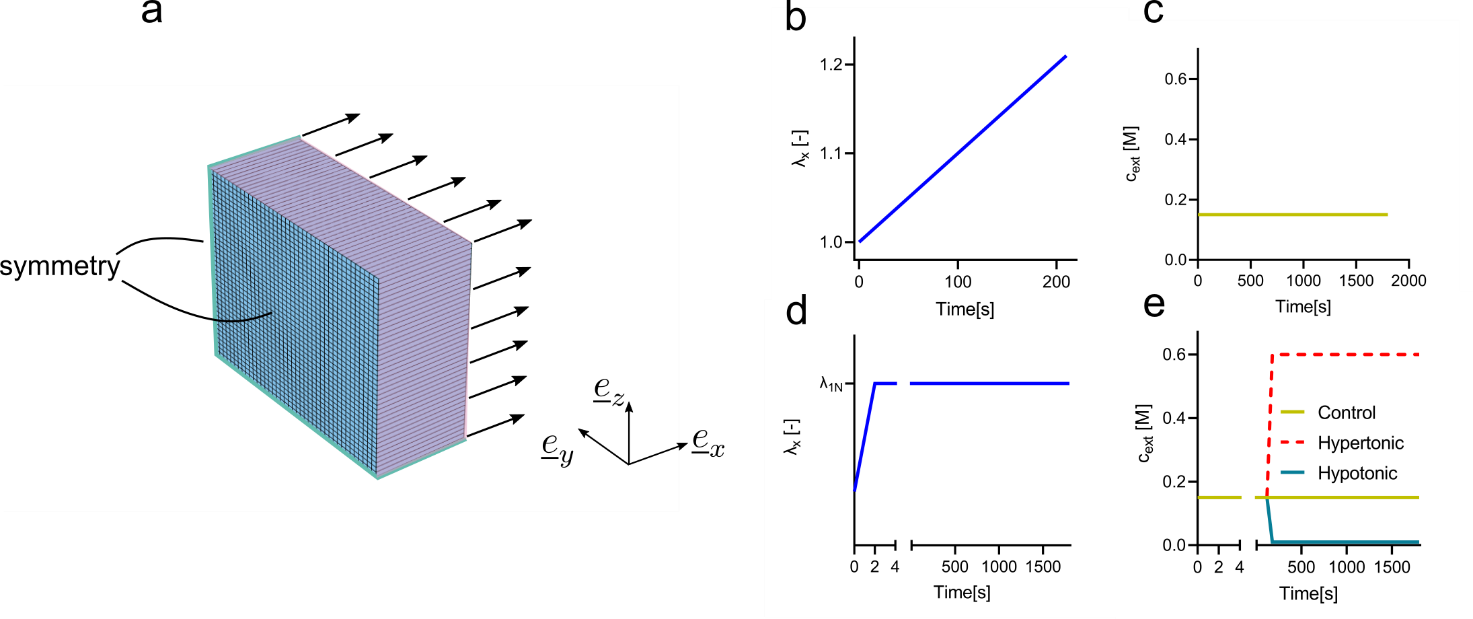


Supplementary Figure 2: (a) Finite element model of uniaxial simulation. with boundary conditions. Boundary conditions on (b) displacement and (c) external bath concentration for monotonic uniaxial experiment. Boundary conditions on (d) force and (e) external bath concentration for relaxation experiment with change of external bath concentration.

The monotonic uniaxial as well as the relaxation experiments are modeled with the finite element model depicted in Supplementary Figure 2. A quarter of the specimen is modeled with symmetry conditions applied on the faces perpendicular to $e_{x}$and $e_{y}$ (indicated). The applied displacement is indicated by arrows. The top and lateral boundary (purple) and the bottom boundary (not visible) can deform freely. A no flow condition is applied on the top surface for ions and fluid flow. Both, ions, and fluid can freely enter the tissue through the bottom and lateral side.

## 2. The strain-generated electrical potential depends on the permeability


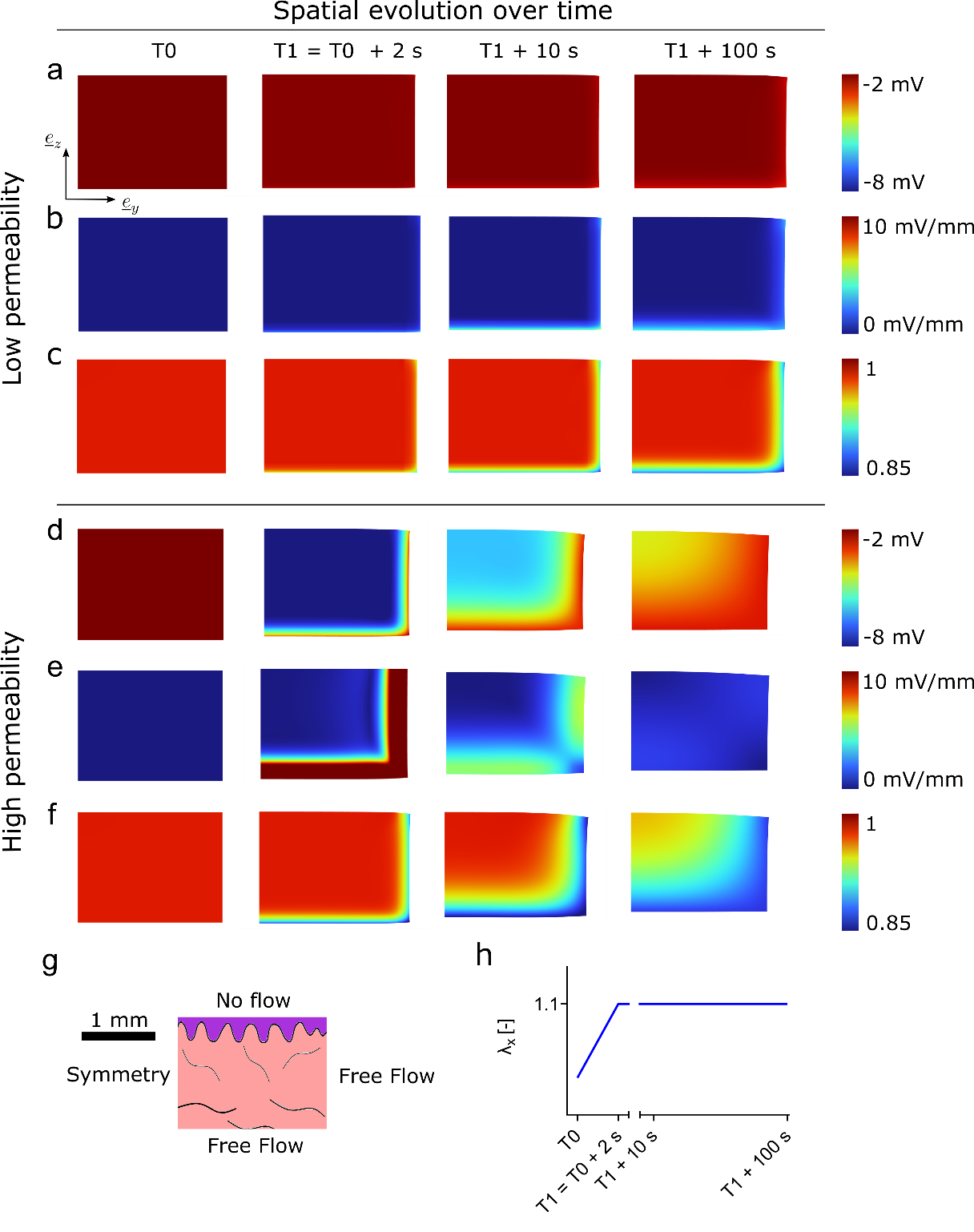


Supplementary Figure 3: The strain generated potential (rows (a) and (d)) and the corresponding magnitude of gradient (rows (b) and (e)) and volume change (rows (c) and (f)) in uniaxial tension relaxation for different time-points (g) orientation of skin and dimension of specimen, (h) protocol applied for uniaxial stretch. The coordinate system indicated in the first graph applies to all images.

A different permeability results in differences in the temporal evolution of the volume change of the tissue and thus affects the electrical potential. Supplementary Figure 3 shows the results of the electrical potential and the magnitude of its gradient over the central cross-section for the same conditions as presented in Figure 9. The simulations presented here were performed with a lower permeability ${k_{0}=10}^{-15}\frac{m^{4}}{\mathrm{Ns}}$ and a higher permeability $k_{0}={10}^{-12}\frac{m^{4}}{\mathrm{Ns}}$ when compared to the results in Figure 9. The electrical potential drops quickly in the inside of the tissue for the high permeability case from -3 mV to -8.0 mV. For the low permeability case the electrical potential almost stays constant. At the beginning (T1) a large gradient exists close to the boundary with magnitudes of up to 30 mV/mm for the high permeability case. The gradient of electrical potential then becomes ‘smoother’ as the tension force is relaxing. Yet, spatial variations in chemical potential remain at 100 s after application of the deformation. While both boundaries approach the new equilibrium potential of -3.5 mV, there is a still a difference of 1 mV and 1.5 mV between the boundary and the center for the high permeability case. High permeability generates higher values of electric field.

## 3. Sensitivity Study of the Model fit to the permeability


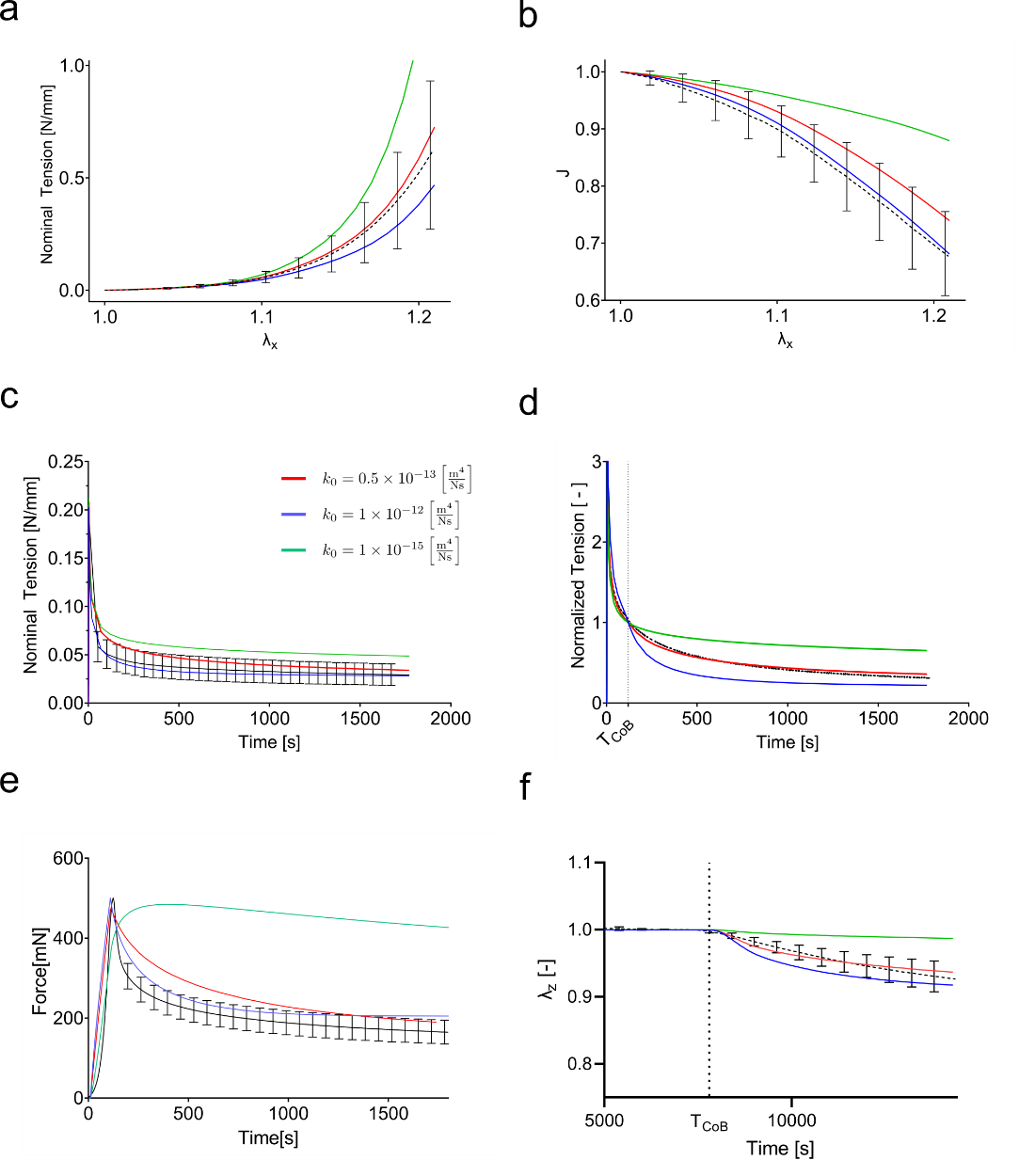


Supplementary Figure 4: Influence of the permeability on (a) nominal tension and (b) volume reduction in uniaxial tension, (c) uniaxial relaxation, (d) dynamics of change of bath from physiological saline to hypertonic saline (4 x physiological), (e) displacement-controlled compression tests, (f) force-controlled compression test with change of bath. The black lines correspond to the experimental data with standard deviation. The model fit is shown in red, and simulations with upper and lower boundaries for permeability are represented in blue and green, respectively.

The effect of alteration of the permeability parameter k_0_ on the mechanical response was investigated by applying the same values of permeability considered for the sensitivity study reported in Supplementary Figure 3, i.e. ${k_{0}=10}^{-15}\frac{m^{4}}{Ns}$ , ${k_{0}=5 \times10}^{-14}\frac{m^{4}}{Ns}$ and ${k_{0}=10}^{-12}\frac{m^{4}}{Ns}$ . Supplementary Figure 4 shows the corresponding results along with the experimental data. The influence of the permeability parameter is visible for all considered load cases. For the relaxation and compression experiments with change of bath, low permeability leads to a dampened mechanical response of the material while large permeability overestimates the tension and stretch decrease, respectively. Consideration of all load cases indicates that the selected value of ${k_{0}=5 \times10}^{-14}\frac{m^{4}}{Ns}$ provides a reasonable agreement across the experimental observations.

## 4. Mesh refinement analysis

The results reported in the paper were obtained with a sufficiently refined mesh. This conclusion is based on a corresponding mesh refinement analysis. On example is reported in this section for the case of uniaxial relaxation, which was then also used to calculate the electrical potential. Results are reported for four different cases: the coarse mesh consisted of 60 elements, increasing to 361 elements for the medium mesh size, and 1369 elements for medium mesh elements in the center of the specimen and finer elements at the boundary of the specimen. Finally, the finest mesh consists of 1600 elements of equal size. To assess the dependence of the solution on the mesh size global and local results are reported in Supplementary Figure 5 for the case of 10% skin is elongated within 2 seconds. Time stepping and solver configurations were equal for all simulations. Nominal tension values (a) are insensitive to meshing in that even the coarsest mesh provides reliable results. The local results were extracted for an element in the middle of the side boundary where gradients are particularly large. Anions concentration and osmotic pressure show convergent results for progressive mesh refinement. As ions concentration correlates with the electric potential and osmotic pressure correlates with the density of fixed charges, similar convergence is obtained for the chemical and electrical potential, demonstrating the reliability of the calculations.


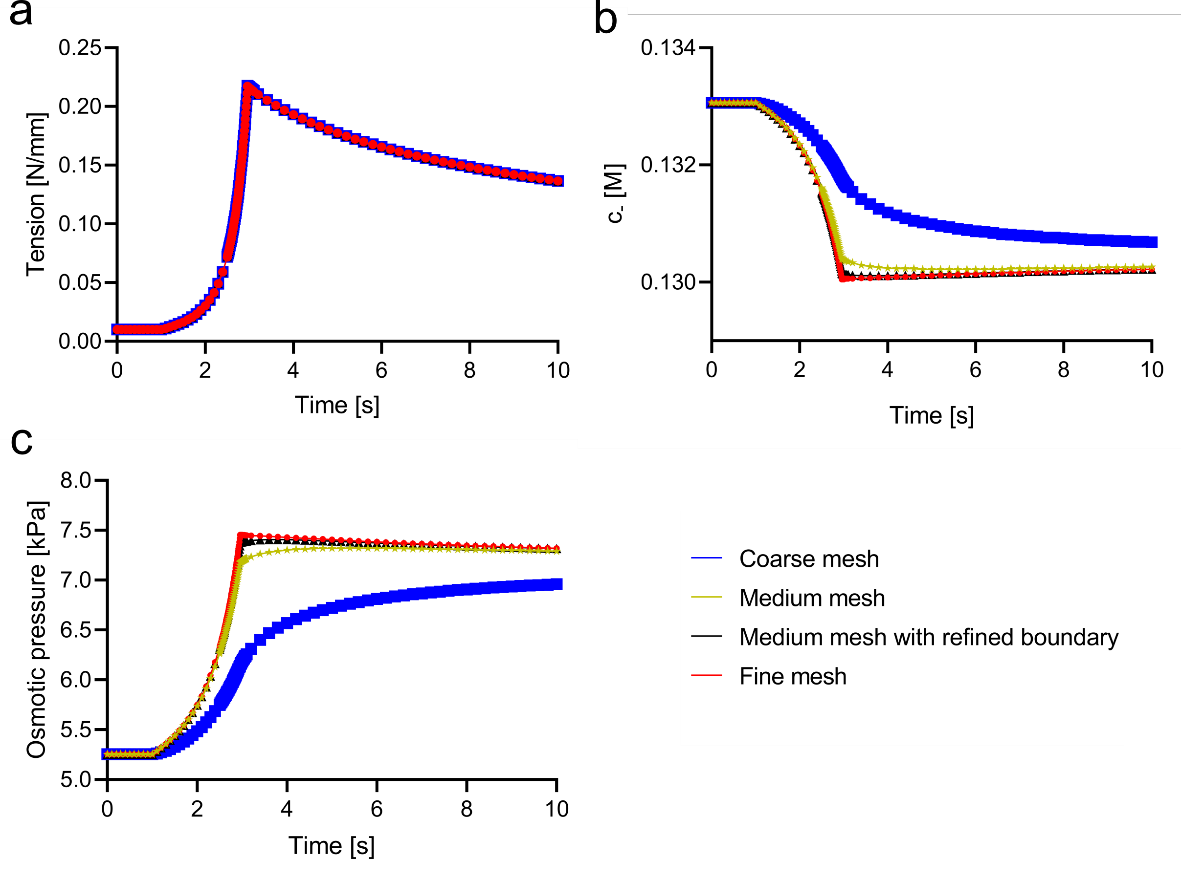


Supplementary Figure 5: (a) Nominal tension, (b) ion concentration and (c) osmotic pressure during the first 10 seconds of a relaxation simulation. Local results are extracted for an element at the side boundary where gradients are particularly large.

# 5. References

Ateshian, G. A., Maas, S., & Weiss, J. A. (2013). Multiphasic finite element framework for modeling hydrated mixtures with multiple neutral and charged solutes. *Journal of Biomechanical Engineering*, *135*(11). https://doi.org/10.1115/1.4024823/370874

Ateshian, G. A., & Weiss, J. A. (2013). Finite Element Modeling of Solutes in Hydrated Deformable Biological Tissues. *Computer Models in Biomechanics*, 231–249. https://doi.org/10.1007/978-94-007-5464-5_17

Ehlers, W., Karajan, N., & Markert, B. (2009). An extended biphasic model for charged hydrated tissues with application to the intervertebral disc. *Biomechanics and Modeling in Mechanobiology*, *8*(3), 233–251. https://doi.org/10.1007/s10237-008-0129-y

Holzapfel, G. A. (2000). *Nonlinear solid mechanics : a continuum approach for engineering*. Wiley. https://www.wiley.com/en-us/Nonlinear+Solid+Mechanics%3A+A+Continuum+Approach+for+Engineering-p-9780471823193

Lanir, Y. (1987). Biorheology and fluid flux in swelling tissues. I. Bicomponent theory for small deformations, including concentration effects. *Biorheology*, *24*(2), 173–187. https://doi.org/10.3233/BIR-1987-24210

Sachs, D., Wahlsten, A., Kozerke, S., Restivo, G., & Mazza, E. (2021). A biphasic multilayer computational model of human skin. *Biomechanics and Modeling in Mechanobiology*, *20*(3), 969–982. https://doi.org/10.1007/s10237-021-01424-w

Sun, D. N., Gu, W. Y., Guo, X. E., Lai, W. M., & Mow, V. C. (1999). A mixed finite element formulation of triphasic mechano-electrochemical theory for charged, hydrated biological soft tissues. *International Journal for Numerical Methods in Engineering*, *45*, 1375–1402. https://doi.org/10.1002/(SICI)1097-0207(19990810)45:10

Wahlsten, A., Pensalfini, M., Stracuzzi, A., Restivo, G., Hopf, R., & Mazza, E. (2019). On the compressibility and poroelasticity of human and murine skin. *Biomechanics and Modeling in Mechanobiology*, 1–15. https://doi.org/10.1007/s10237-019-01129-1

Wilson, W., Van Donkelaar, C. C., & Huyghe, J. M. (2005). A Comparison Between Mechano-Electrochemical and Biphasic Swelling Theories for Soft Hydrated Tissues. *Journal of Biomechanical Engineering*, *127*(1), 158–165. https://doi.org/10.1115/1.1835361
